# An Open Label, Adaptive, Phase 1 Trial of High‐Dose Oral Nitazoxanide in Healthy Volunteers: An Antiviral Candidate for SARS‐CoV‐2

**DOI:** 10.1002/cpt.2463

**Published:** 2021-11-13

**Authors:** Lauren E. Walker, Richard FitzGerald, Geoffrey Saunders, Rebecca Lyon, Michael Fisher, Karen Martin, Izabela Eberhart, Christie Woods, Sean Ewings, Colin Hale, Rajith K. R. Rajoli, Laura Else, Sujan Dilly‐Penchala, Alieu Amara, David G. Lalloo, Michael Jacobs, Henry Pertinez, Parys Hatchard, Robert Waugh, Megan Lawrence, Lucy Johnson, Keira Fines, Helen Reynolds, Timothy Rowland, Rebecca Crook, Emmanuel Okenyi, Kelly Byrne, Pavel Mozgunov, Thomas Jaki, Saye Khoo, Andrew Owen, Gareth Griffiths, Thomas E. Fletcher, the AGILE platform

**Affiliations:** ^1^ University of Liverpool Liverpool UK; ^2^ Liverpool University Hospitals NHS Foundation Trust Liverpool UK; ^3^ Southampton Clinical Trials Unit University of Southampton Southampton UK; ^4^ Liverpool School of Tropical Medicine Liverpool UK; ^5^ MRC Biostatistics Unit University of Cambridge Cambridge UK

## Abstract

Repurposing approved drugs may rapidly establish effective interventions during a public health crisis. This has yielded immunomodulatory treatments for severe coronavirus disease 2019 (COVID‐19), but repurposed antivirals have not been successful to date because of redundancy of the target *in vivo* or suboptimal exposures at studied doses. Nitazoxanide is a US Food and Drug Administration (FDA) approved antiparasitic medicine, that physiologically‐based pharmacokinetic (PBPK) modeling has indicated may provide antiviral concentrations across the dosing interval, when repurposed at higher than approved doses. Within the AGILE trial platform (NCT04746183) an open label, adaptive, phase I trial in healthy adult participants was undertaken with high‐dose nitazoxanide. Participants received 1,500 mg nitazoxanide orally twice‐daily with food for 7 days. Primary outcomes were safety, tolerability, optimum dose, and schedule. Intensive pharmacokinetic (PK) sampling was undertaken day 1 and 5 with minimum concentration (C_min_) sampling on days 3 and 7. Fourteen healthy participants were enrolled between February 18 and May 11, 2021. All 14 doses were completed by 10 of 14 participants. Nitazoxanide was safe and with no significant adverse events. Moderate gastrointestinal disturbance (loose stools or diarrhea) occurred in 8 participants (57.1%), with urine and sclera discoloration in 12 (85.7%) and 9 (64.3%) participants, respectively, without clinically significant bilirubin elevation. This was self‐limiting and resolved upon drug discontinuation. PBPK predictions were confirmed on day 1 but with underprediction at day 5. Median C_min_ was above the *in vitro* target concentration on the first dose and maintained throughout. Nitazoxanide administered at 1,500 mg b.i.d. with food was safe with acceptable tolerability a phase Ib/IIa study is now being initiated in patients with COVID‐19.

**Study Highlights**

**WHAT IS THE CURRENT KNOWLEDGE ON THE TOPIC?**

☑ Nitazoxanide is an anti‐parasitic medication licensed by the US Food and Drug Administration (FDA) at standard dosing (500 mg b.i.d.) with an established safety profile. Antiviral activity has been demonstrated for numerous viruses with *in vitro* data demonstrating activity against severe acute respiratory syndrome‐coronavirus 2 (SARS‐CoV‐2). No steady‐state pharmacokinetic (PK) data are available at higher doses or in coronavirus disease 2019 (COVID‐19) but physiologically‐based PK modeling has indicated a 1500 mg b.i.d. regimen will achieve required SARS‐CoV‐2 plasma effective concentration 90% (EC_90_) concentrations across the dosing period.

**WHAT QUESTION DID THIS STUDY ADDRESS?**

☑ Is high‐dose nitazoxanide safe and well‐tolerated in healthy individuals and can it achieve and maintain plasma antiviral concentrations predicted to be sufficient to prevent maturation of the SARS‐CoV‐2 spike protein and therefore drive antiviral efficacy?

**WHAT DOES THIS STUDY ADD TO OUR KNOWLEDGE?**

☑ Plasma concentrations of tizoxanide, the major circulating form of nitazoxanide, are sufficient to maintain the *in vitro* derived EC_90_ and can be safely achieved in healthy individuals. The 1,500 mg b.i.d. dose has acceptable tolerability, with mild gastrointestinal side effects in healthy volunteers.

**HOW** **MIGHT THIS CHANGE CLINICAL PHARMACOLOGY OR TRANSLATIONAL SCIENCE?**

☑ This phase Ia study precedes a seamless phase Ib/IIa evaluation of high dose nitazoxanide in mild/moderate COVID‐19 within the AGILE platform. It has provided key information on the PK profile and tolerability at higher doses that supports its evaluation in patients with COVID‐19 and potential use as an antiviral in other diseases. These doses will give the maximal opportunity to achieve antiviral concentrations for SARS‐CoV‐2 but the efficacy of nitazoxanide for COVID‐19 can only be determined in subsequent trials in patients.

Since the emergence of coronavirus disease 2019 (COVID‐19) in Wuhan, China, in 2019, numerous treatment candidates have been tested in late‐phase clinical trials. To date, limited therapeutic options^1^ exist, such as steroids and IL‐6 receptor blockers (tocilizumab and sarilumab) as immunodulators in severe disease, and emerging signals of efficacy of antiviral monoclonal antibody therapies. Repurposing of approved drugs is in principle the fastest way to establish interventions during an urgent public health crisis. This has yielded interventions for severe COVID‐19, but efforts to establish repurposed antiviral interventions have not been successful to date. Reasons for failure are multifaceted and relate to the pharmacokinetics (PKs) and pharmacodynamics of the candidate antiviral drugs. In terms of PKs, most putative repurposing drugs that have been studied preclinically are not expected to reach systemic antiviral concentrations at the approved dose and schedule.^2^ Redundancy of the expected mechanism of action *in vivo* has also been reported, which was driven by inadequacy of the *in vitro* model used to demonstrate activity. For example, the primary mechanism of antiviral activity for hydroxychloroquine in Vero cells involved a process for viral entry, which is secondary *in vivo*. As such, hydroxychloroquine activity is mitigated in animal models and in cells expressing TMPRSS2.^3^, ^4^

Nitazoxanide is a thiazolide US Food and Drug Administration (FDA) approved antiparasitic medicine used for the treatment of cryptosporidiosis and giardiasis^5^ and has also reported activity against anaerobic bacteria, protozoa, and several other viruses.^6^ Rapid deacetylation of nitazoxanide in blood means that the major systemic species of the drug *in vivo* is tizoxanide. Tizoxanide has been shown to exhibit similar *in vitro* inhibitory activity to nitazoxanide for rotaviruses,^7^ hepatitis B and C viruses,^8^, ^9^ coronaviruses other than severe acute respiratory syndrome‐coronavirus 2 (SARS‐CoV‐2) and noroviruses.^10^, ^11^ It has also demonstrated *in vitro* activity against influenza viruses^12^, ^13^ and in a phase IIb/III trial in uncomplicated influenza, nitazoxanide demonstrated a reduction in symptoms and viral shedding at a dose of 600 mg twice‐daily compared with placebo,^14^ despite these doses providing systemic concentrations that are only expected to remain above the influenza *in vitro* target for a fraction of the dosing interval.^2^ Similarly, several recent small clinical trials have indicated some antiviral and clinical benefits of nitazoxanide but at doses that are not expected to maintain concentrations above the *in vitro* antiviral target for the full dosing interval.^15^, ^16^, ^17^ Although this is encouraging, higher doses and combinations are likely to be ultimately needed to maximize the antiviral activity and mitigate the risk of emergence of drug resistance.^18^, ^19^ The antiviral mechanism of action of nitazoxanide against influenza involves an impact upon post‐translational modification and maturation of hemagglutinin,^20^ and a similar mechanism involving the SARS‐CoV‐2 spike protein was also recently reported.^21^

Other potential benefits of nitazoxanide in COVID‐19 may derive from its impact upon the innate immune response that potentiates the production of type 1 interferons^13^, ^22^ and bronchodilation of the airways through inhibition of TMEM16A ion channels.^23^ Moreover, a recent study indicated that drugs which inhibit TMEM16, like nitazoxanide, block the SARS‐CoV‐2 spike protein‐mediated syncytia formation via a mechanism independent of their antiviral activity.^24^ At conventional doses of 500 mg twice daily, nitazoxanide achieves C_trough_ plasma concentrations close to the *in vitro* defined target concentration for SARS‐CoV‐2,^25^ and exhibits antiviral activity in cell types that recapitulate *in vivo* mechanism of replication.^15^ The highest nitazoxanide dose reported to date in COVID‐19 clinical trials is 1,000 mg twice‐daily, utilized in combination with another agent.

Published physiologically‐based PK (PBPK) modeling, validated against tizoxanide PKs after single doses ranging between 500 and 4,000 mg as well as twice daily doses of 0.5 and 1 g, indicated doses above those already approved may provide C_trough_ above the antiviral target in the majority of patients.^2^ Modeling estimated that 1,400 mg twice‐daily or 900 mg 3‐times daily will provide pulmonary exposures for the entire dosing interval above the reported *in vitro* effective concentration 90% (EC_90_) for SARS‐CoV‐2 in over 90% of the population. Nitazoxanide has an established safety record in humans and studies showed tolerability of single oral doses up to 4,000 g with minimal gastrointestinal (GI) side effects. In a separate study involving 16 healthy men, doses of either 500 mg twice‐daily or 1,000 mg twice‐daily for 7 days, the 500 mg dose was well‐tolerated with only mild adverse events not differing significantly from the placebo.^26^ The 1,000 mg twice‐daily dose was associated with an increased frequency of GI side effects, primarily diarrhea and abdominal discomfort, but no significant changes were noted in the safety parameters and laboratory tests.

The combination of a long‐established safety track record along with its demonstrated *in vitro* activity against coronaviruses, including SARS‐CoV‐2, and its availability from global generic manufacturers at extremely low cost, makes nitazoxanide an attractive therapeutic option for dose escalation and repurposing. As such, it was prioritized for evaluation at higher doses within the AGILE clinical trial platform, as a potential therapeutic for mild/moderate disease.

## METHODS

### Study design

This was a single‐center, open label, adaptive, phase I trial in healthy adult participants (NCT04746183). This was undertaken as a candidate specific trial within AGILE; a phase Ib/IIa platform trial for evaluating new therapies for COVID‐19 (www.agiletrial.net), comprising an over‐arching Master Protocol^27^ under which sits candidate‐specific trials evaluating specific compounds. The study protocol was reviewed and approved by the UK Medicines and Healthcare products Regulatory Agency (MHRA) and West Midlands Edgbaston Research Ethics Committee. The study was coordinated by the National Institute for Health Research (NIHR) Southampton Clinical Trials Unit with participants recruited into the NIHR Royal Liverpool and Broadgreen Clinical Research Facility (UK).

### Participants

**Figure 1:**
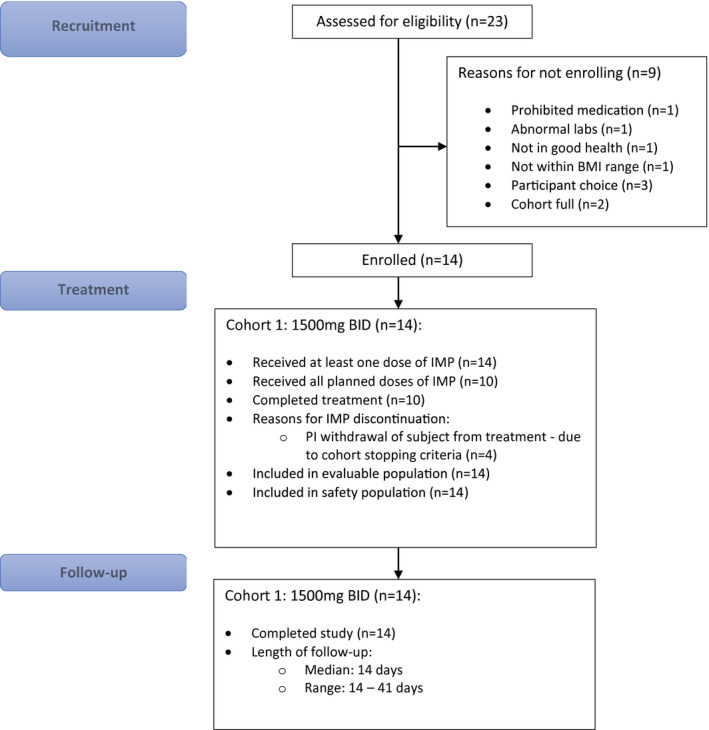
CONSORT diagram. BMI, body mass index; PI, principal investigator. [Colour figure can be viewed at wileyonlinelibrary.com]

Eligible participants included healthy adult men and nonpregnant and nonlactating women between 18 and 75 years of age. Women of childbearing potential and men (who are sexually active with female partners of childbearing potential) were required to use two effective methods of contraception, one of which should be highly effective, throughout the study and for 50 days (women) and 100 days (men) thereafter. Participants were included if they were confirmed to be healthy in the absence of any clinically significant cardiovascular, respiratory, GI, neurological, psychiatric, metabolic, endocrine, renal, hepatic, hematological, or other major disorder. Specific exclusion criteria included: pregnancy or currently lactating, being in receipt of any medication, including St. John’s Wort, known to chronically alter drug absorption or elimination within 30 days prior to first dose administration. In particular, owing to the high protein binding of tizoxanide; warfarin, phenytoin, amiodarone, and intravenous chemotherapy were specifically prohibited within 30 days or 5 half‐lives (whichever was longer) of first dose administration and up to the end of the study. Participants in receipt of any prescribed medication that required dose alteration or any nonprescribed systemic or topical medication, herbal remedy, or vitamin/mineral supplementation within 14 days prior to the first dose administration (unless, in the opinion of the investigator, it would not interfere with study procedures or compromise safety) were specially excluded. Additionally, those with any clinically significant allergy or those that had previously received nitazoxanide or its constituent parts within 3 months of receiving first dose. All participants provided written informed consent before enrollment. The full list of inclusion and exclusion criteria can be found in **Table** **S1**. The flow of participants is outlined in **Figure** **1**.

### Model refinement based upon phase I steady‐state pharmacokinetic data

The PK parameters used for the model construction were obtained from a previous publication (**Table** **S2**).^28^ The initial prediction was made using this PBPK model that was validated against steady‐state PKs at 500 and 1,000 mg b.i.d. and the trial outcome demonstrated greater accumulation of drug at steady‐state for 1,500 mg b.i.d., with higher C_trough_ than was predicted from previous studies with 500 and 1,000 mg b.i.d.^26^ Because nitazoxanide has been proposed as a broad‐spectrum antiviral and also has unrelated suggested utility in several noncommunicable diseases, the model was refined so as to be valuable for future dose predictions. For this, the absorption rate constant (*K*_a_) was adjusted to fit the steady‐state 1,500 mg b.i.d. PKs as follows:

(1) $$\text{ka}=\text{ka}*\left(1‐0.05*\text{nod}\right)$$

where nod is an integer that stands for the number of doses at time t.

The above equation evaluates the value of *K*_a_ at every time point as the number of doses (nod) increase until it reaches 4 and then remains constant until the end of the simulation period.

### Dosing

Eligible participants received 1,500 mg nitazoxanide orally with food, twice‐daily for 7 days. Dosing with food was undertaken on the inpatient unit on day 1 and day 5; the remaining dosing was administered by the participants at home with instruction to eat within 30 minutes of dosing. In this study, we had predefined 3 possible dosing strategies for nitazoxanide: (1) 1,500 mg 12 hourly (“b.i.d. dosing”), which was predicted by our PK models to achieve the desired target concentrations, plus a further 2 regimens of (2) 1,000 mg 8 hourly (“t.i.d. dosing”), and (3) 1,500 mg in the morning, 2,000 mg in the evening (“asymmetric dosing”). A Safety Review Committee (SRC) was planned to review the initial regimen (1,500 mg b.i.d.) and if deemed a failure because of toxicity or failure to achieve target plasma concentrations could recommended progression to the 1,000 mg t.i.d. and/or asymmetric dose cohorts. If the SRC considered a regimen as safe with an achievement of target plasma concentrations, they could recommend its further investigation in a future phase Ib/IIa trial in patients with COVID‐19.

### Study procedures

Viral polymerase chain reaction swab screening to exclude COVID‐19 was performed for all participants 48‐hours prior to admission to the unit. General physical examination, serum chemistry, and hematology sampling were performed at screening, 48 hours prior to dosing, day 1 baseline, and 72 and 120 hours postdose followed by the final study visit at day 14 post‐first dose. Urinalysis was performed at screening, 48 hours predose, 72 hours postdose, followed by the final study visit at day 14 post‐first dose. Adverse events (AEs) were collected from the time of start of the first dose and throughout the study period. For GI tolerance, the safety focus point was stool frequency and consistency assessed according to the Bristol Stool Chart (BSC).

### Outcomes

The primary outcome for this study was to assess safety, tolerability, optimum dose, and dosing schedule of nitazoxanide in healthy participants through the following parameters: AEs, general physical examination, general safety assessments, including electrocardiogram (ECG), vital signs, clinical laboratory analysis, including urinalysis, hematology, and serum chemistry. Because GI intolerance was anticipated at the high doses of nitazoxanide used, participants were asked to fill in a BSC for the duration of assessment. The primary end point for this trial was safety and tolerability defined by AE frequency and severity of nitazoxanide in healthy volunteers assessed over 10 days.

Unacceptable safety and tolerability parameters were defined as:

- A treatment emergent serious AE (SAE) in one subject that was considered by the investigator to be related to the study drug.
- Two or more AEs, the clinical severity of which was graded by the investigator as 3 (severe), 4 (life threatening), or 5 (resulting in death).
- Two or more participants in a cohort required withdrawal due to elevated alanine aminotransferase (ALT) levels:
  ο Asymptomatic elevation in ALT or aspartate aminotransferase to more than 3‐times the upper limit of normal (ULN), confirmed by a repeat measurement within 48 to 72 hours, accompanied by either direct bilirubin level more than or equal to 2‐times the ULN or International Normalized Ratio (INR) more than or equal to 1.5, confirmed with repeat measurement within 48–72 hours.
  ο Symptomatic elevation in ALT to more than 3‐times the ULN.
- Significant diarrhea, defined as 7 or more episodes, rated greater than or equal to five on the BSC, in 1 day for 2 consecutive days was seen in 2 or more participants in a group.

Additional participant withdrawal criteria included:

- Any clinically relevant signs or symptoms that, in the opinion of the investigator, warrant participant withdrawal.
- QTcB prolongation to > 500 milliseconds or a rise in QTcB value of > 60 milliseconds (whichever was lower), observed on triplicate ECGs, compared with the baseline mean QTcB.
- Positive urine drugs of abuse screen, alcohol breath test, or pregnancy test result.

### Pharmacokinetic analysis

Intensive plasma PK sampling for tizoxanide and tizoxanide glucuronide was undertaken on day 1 and day 5. Blood samples were collected predose, 0.5, 1, 1.5, 2, 3, 4, 6, 8, and 12 hours after first dose administration. Additional C_trough_ PK samples were obtained at 12‐hours postdose on days 3 and 7. Tizoxanide and tizoxanide glucuronide concentrations in plasma were measured using a validated liquid chromatography tandem mass spectrometry method. In brief, analytes were extracted from plasma by protein precipitation with 0.1% formic acid in acetonitrile, centrifuged, and diluted in a reconstitution solution (5 mM ammonium formate: acetonitrile, 50:50 v/v) before injection onto the high‐performance liquid chromatography column. Deuterated (D_4_) internal standards were used and chromatographic separation was achieved using a reverse phase C_18_ column. The calibration range was linear between 50 and 45,000 ng/mL for both analytes. As per protocol, PK success in the trial was defined as median C_trough_ values above the 1.43 mg/L derived from the *in vitro* EC_90_ against SARS‐CoV‐2.

The combined parent tizoxanide and tizoxanide‐glucuronide metabolite dataset was fitted with a parent‐metabolite PK model using one‐compartment disposition for both tizoxanide and tizoxanide‐glucuronide, with first order absorption/appearance for tizoxanide. The model is similar to previous parent‐metabolite PK models applied for example to rifapentine^29^ and a schematic and rate equations are provided in **Figure** **S1**. In the absence of definitive mass balance data for fraction of total parent drug clearance accounting for the formation route of the glucuronide metabolite, apparent clearance (CL_met_), and V_met_ parameters were estimated for the metabolite. Fitting of this PK model to the observed plasma concentration data was carried out in the R programming environment (version 4.0.3)^30^ making use of the Pracma library^31^ and lsqnonlin function for nonlinear regression, treating the dataset from all participants as a naïve pool. When further data become available at the current dose, this PK model can be applied using a nonlinear‐mixed effects population approach for characterization of interindividual variability and potential covariate relationships with PK parameters.

In previously published work, a PBPK model was validated for nitazoxanide using Simbiology (MATLAB R2019a; MathWorks, Natick, MA, USA) and used to inform dose selection for the current trial.^28^ Therefore, a comparison was made between the observed PKs in the trial and the *a priori* validated PBPK model prediction.

### Statistical analysis

We utilized a Bayesian adaptive design to support decision making in this phase Ia study. Details are provided in **Supplementary Material**. Briefly, there was uncertainty about the order of some of the dosing strategies, with respect to their toxicity. To enable us to relax the assumption of monotonicity of toxicity, we used the Partial Ordering Continual Reassessment Method (POCRM) design proposed by Wages *et al*.^32^ adjusted to the AGILE setting.

The POCRM utilizes a simple, one‐parameter logistic dose‐toxicity model, which describes the relationship between dose‐limiting toxicities (DLTs) within 10 days of initiating treatment and treatment dose (1,500 mg b.i.d., 1,000 mg t.i.d., and asymmetric dosing) and includes the uncertainty about orderings in the working model itself. For each possible ordering, the POCRM fits a continual reassessment method model and finds the posterior probability of each ordering being the true ordering. The model that is deemed the most likely one given the data observed is used for the escalation/de‐escalation decisions. The prior distributions for the POCRM were calibrated to maximize the proportion of correct selection under the range of possible orderings and dose‐toxicity scenarios within each ordering, where each dose strategy considered in the study was the optimum one.

At the end of the cohort, the SRC reviewed all available safety data, including at least 10 days data for each participant in the cohort, including data on AEs, vital signs, ECGs, and clinical laboratory evaluations, as well as any emerging data from other studies. Following SRC review, recommendations could be to de‐escalate, escalate, remain at the same dose, or continue to phase Ib. A dose was deemed to be unsafe if there was a ≥ 25% chance that treatment was associated with a > 20% risk of DLTs at day 10. The model recommended the next dose‐level according to which level is the most likely to correspond to the DLT rate of 5–15%. However, the SRC made the ultimate decision whether to accept that the current dose was safe and met the target median C_trough_ value of 1.43 mg/L.

All analyses are reported according to CONSORT 2010 and International Conference on Harmonization (ICH) E9 guidelines on Statistical Principles in Clinical Trials. All enrolled participants were included in both the evaluable population and the safety population for analysis. Statistical analysis was undertaken in SAS version 9.4, STATA version 16, and R version 4.0.2. Baseline demographics and AEs are summarized using descriptive statistics. The estimated DLT rates for each dose strategy and equal‐tail 95% credible intervals taken from the model of the most likely ordering in **Table** **1**. For active doses, we also present the probability that the DLT rate falls within 5–15% (a predetermined acceptable target range for toxicity) and the probability of at least 20% toxicity (deemed as unacceptably toxic).

**Table 1:** Estimated toxicity for nitazoxanide up to day 10 from the POCRM model (for ordering 3)

Dose level	Estimated DLT rate (95% Credible interval)	Target dose probability, 5–15% DLT rate	Probability of being overly toxic, toxicity > 20%
1,500 mg b.i.d.	2.1% (0.0–14.4%)	11.8%	0.9%
1,000 mg t.i.d.	0.2% (0.0–2.1%)	0.6%	0.0%
Asymmetric	9.0% (0.0–36.2%)	26.6%	15.2%

DLT, dose‐limiting toxicity; POCRM, Partial Ordering Continual Reassessment Method.

The sample size was flexible, based on the need for the study to adapt to accruing safety data. Simulations to assess model operating characteristics and to calibrate priors assumed 3 possible orderings and the same three dose strategies within each ordering, with cohorts of size 12 capped at a total of 36 participants.

## RESULTS

Between February 18 and May 11, 2021, 14 healthy volunteers received at least one dose of nitazoxanide (**Table** **S3**.) Of 14 participants dosed, 10 of 14 (71.4%) completed 7 full days of dosing (14 doses). Four participants discontinued dosing as a result of an artifactual QTcB prolongation occurring in one participant, which led to suspension of dosing in the entire cohort. Four of 14 participants discontinued dosing early; 2 of 4 discontinued after 6 full days (12/14 doses), and the remaining 2 of 4 discontinued after 1 full day (2/14 doses). All 14 participants were included in the safety and PK analyses. The flow of trial participants is shown in **Figure** **1**. The demographics and clinical characteristics at baseline are summarized in **Table** **2**.

**Table 2:** Participant demographics and characteristics

Characteristics	Statistic	Nitazoxanide (1,500 mg twice‐daily, *n* = 14)
Age	Median (range)	25 (19–53)
Sex	Male/female %	35.7%/64.3%
Ethnicity	White‐English/Welsh/Scottish/Northern Irish/British	9 (64.3%)
	Any other White background	3 (21.4%)
	Any other Asian background	2 (14.3%)
Body weight (kg)	Median (range)	65.6 (61.2–96.3)
BMI (kg/m^2^)	Median (range)	23.9 (19.3–30.8)

BMI, body mass index.

### Safety results

Nitazoxanide was safe with acceptable tolerance at 1,500 mg twice daily for 7 days with no significant AEs (**Table** **3**). Moderate GI disturbance (sufficient to interfere with daily activities) was seen in three participants (21.4%) with a further eight participants (57.1%) experiencing mild GI symptoms (covering a spectrum of nausea, bloating, constipation, diarrhea or loose stools, and/or abdominal pain). Yellow discoloration of the urine and sclera was observed in 12 (85.7%) and 9 (64.3%) participants, respectively, without clinically significant elevation in bilirubin. This was self‐limiting and resolved upon discontinuation of the drug. No grade 3 or 4 AEs were documented.

**Table 3:** AEs and SAEs

Adverse event (all grades 1 or 2)	Nitazoxanide (1,500 mg b.i.d.) (*n* = 14)
Urine discoloration	12 (85.7%)
Yellow sclera	9 (64.3%)
Nausea	7 (50.0%)
Abdominal pain	6 (42.9%)
Diarrhea	5 (35.7%)
Loose stools	3 (21.4%)
Myalgia	3 (21.4%)
Bloating	2 (14.3%)
Headache	2 (14.3%)
Right corneal irritation	1 (7.1%)
Constipation	1 (7.1%)
Flatulence	1 (7.1%)
Fever	1 (7.1%)
Flu like symptoms	1 (7.1%)
Loss of appetite	1 (7.1%)
Non‐cardiac chest pain	1 (7.1%)
Fall	1 (7.1%)
Umbilical hernia	1 (7.1%)
Dizziness	1 (7.1%)
Lethargy	1 (7.1%)
Dysuria	1 (7.1%)
Irregular menstruation	1 (7.1%)
Semen discoloration	1 (7.1%)
Vaginal discharge	1 (7.1%)
Laryngeal inflammation	1 (7.1%)
Blister on abdomen	1 (7.1%)
Hypotension	1 (7.1%)
Abdominal gas (rumbling)	1 (7.1%)
Elevated creatine kinase	1 (7.1%)
Heavy sensation to eyes	1 (7.1%)
Leg pain/stiffness	1 (7.1%)
Mouth ulcer	1 (7.1%)
Musculoskeletal pain (elbow)	1 (7.1%)
Musculoskeletal pain (hip)	1 (7.1%)
Other – stomach cramps	1 (7.1%)
Rash	1 (7.1%)
Reduced appetite	1 (7.1%)
Shortness of breath	1 (7.1%)
Skin discoloration	1 (7.1%)
Stye (right eye)	1 (7.1%)
Yellow semen	1 (7.1%)
SAE, grade 3 Unplanned hospitalization secondary to electrocardiogram QT corrected interval prolonged	1 (7.1%)

AE, adverse event; SAE, serious adverse event.

Minor, self‐resolving, postdose elevation in creatine kinase (CK) was identified in 5 of 14 participants (maximum 869 U/L at D5 postdose; Table S4). One of the participants reported mild localized myalgia without muscle tenderness at D5 (CK 508 U/L), which was recorded as an AE. All other elevations were not considered clinically significant.

In one participant, tachycardia (heart rate 112 bpm) developed shortly after the second dose administration on day 1, with an ECG recorded 7 minutes following the second dose showing a corrected QTcB (using Bazett’s formula) of 468 msec, an apparent increase of 73.5 msec above the mean baseline (394.5 msec; **Figure** **S2**). Treatment was discontinued in all being dosed. Following overnight admission for observation and serial ECGs (SAE; **Table** **3**), an apparent prolongation recurred at 5 hours and 44 minutes following the second dose, 460 msec (65.5 msec above baseline mean, heart rate 87 bpm) and resolved fully thereafter. Independent cardiology review of all ECGs with manual QT and QTcB calculations confirmed an artifactual QTcB increase due to the presence of a prominent U‐wave fused with the end of the T wave, causing the ECG machine to calculate the QU interval instead of the QT interval. Following formal review by the trial SRC and in consultation with the UK medicines regulator, the trial (which had been temporarily suspended) was reopened at the same dose level with replacement of 2 participants who discontinued dosing after only 1 full day. Additional ECGs were obtained at 14 hours post first‐dose on day 1 and day 5 for subsequent participants, with no significant changes observed.

### An updated PBPK model to incorporate changes in steady‐state pharmacokinetics at higher doses

**Figure** **S3** shows an updated PBPK model for forward prediction of steady‐state PKs of tizoxanide. The predicted PK parameters – C_max_, C_trough_, and area under the curve (AUC) had a ratio < 2 against the observed data, as shown in the **Table** **S5**. The observed PKs of tizoxanide at 1,500 mg follow flip‐flop kinetics, the additional equation (Eq. 1) to the absorption phase therefore delays the T_max_ and increases the C_trough_ from day 2 onward. This addition improved the overall prediction of the steady‐state PK with little difference between observed and predicted values. High data variability across individuals due to limited sample size and dosing and/or sampling differences in the observed data may contribute to PK differences between observed and simulated data for C_trough_ at 48 hours and the T_max_ on day 5.

### Pharmacokinetics and modeling

**Figure 2:**
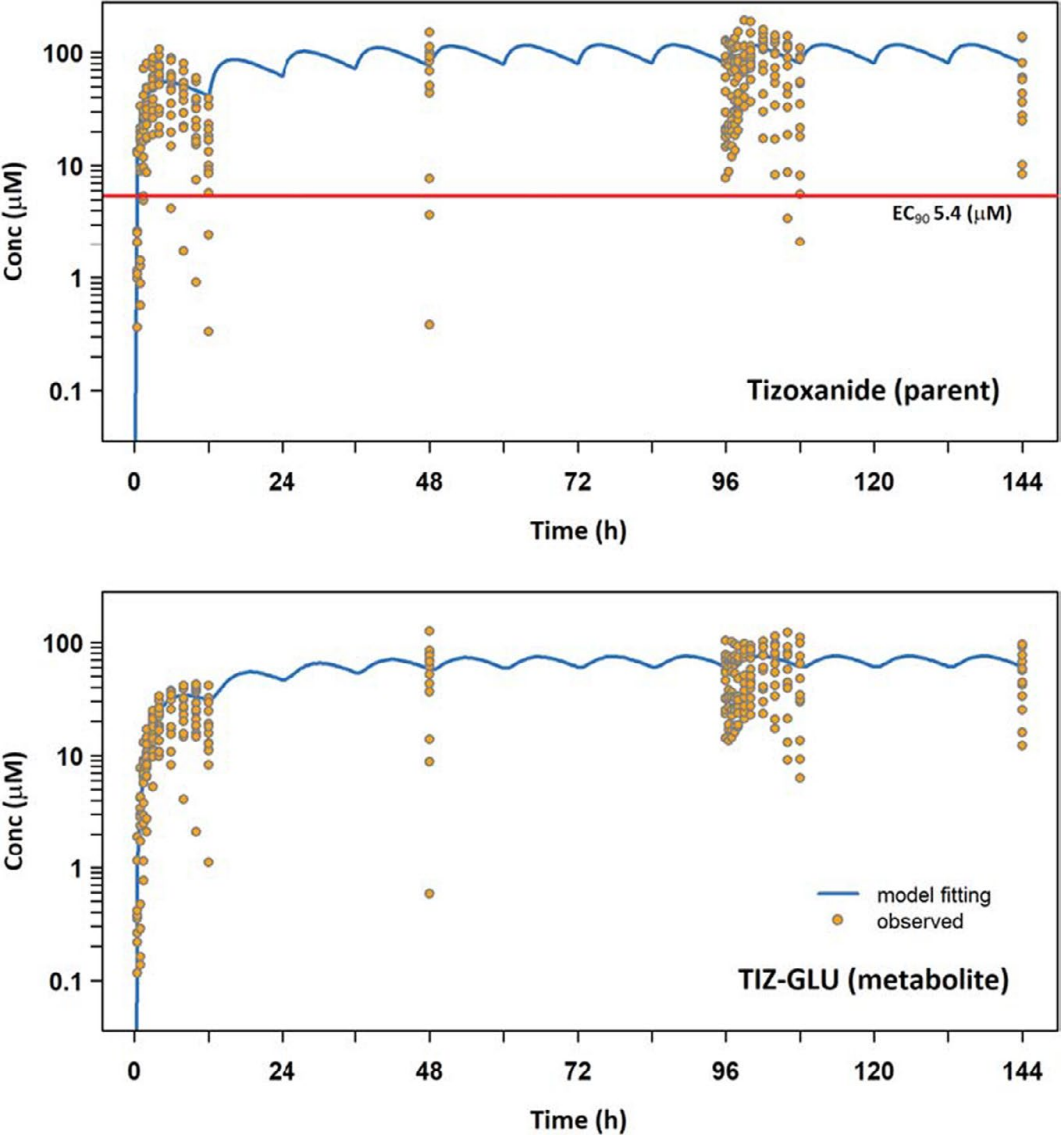
Simultaneous one‐compartment, first order absorption parent‐metabolite PK model fitting to naïve pooled tizoxanide (parent) and tizoxanide‐glucuronide (metabolite) plasma concentration data. The red line represents the *in vitro* derived EC_90_ against SARS‐CoV‐2 (1.43 mg/L). Conc, concentration; EC_90_, effective concentration 90%; GLU, glucuronide; PK, pharmacokinetic; SARS‐CoV‐2, severe acute respiratory syndrome‐coronavirus 2; TIZ, tizoxanide. [Colour figure can be viewed at wileyonlinelibrary.com]

**Figure 3:**
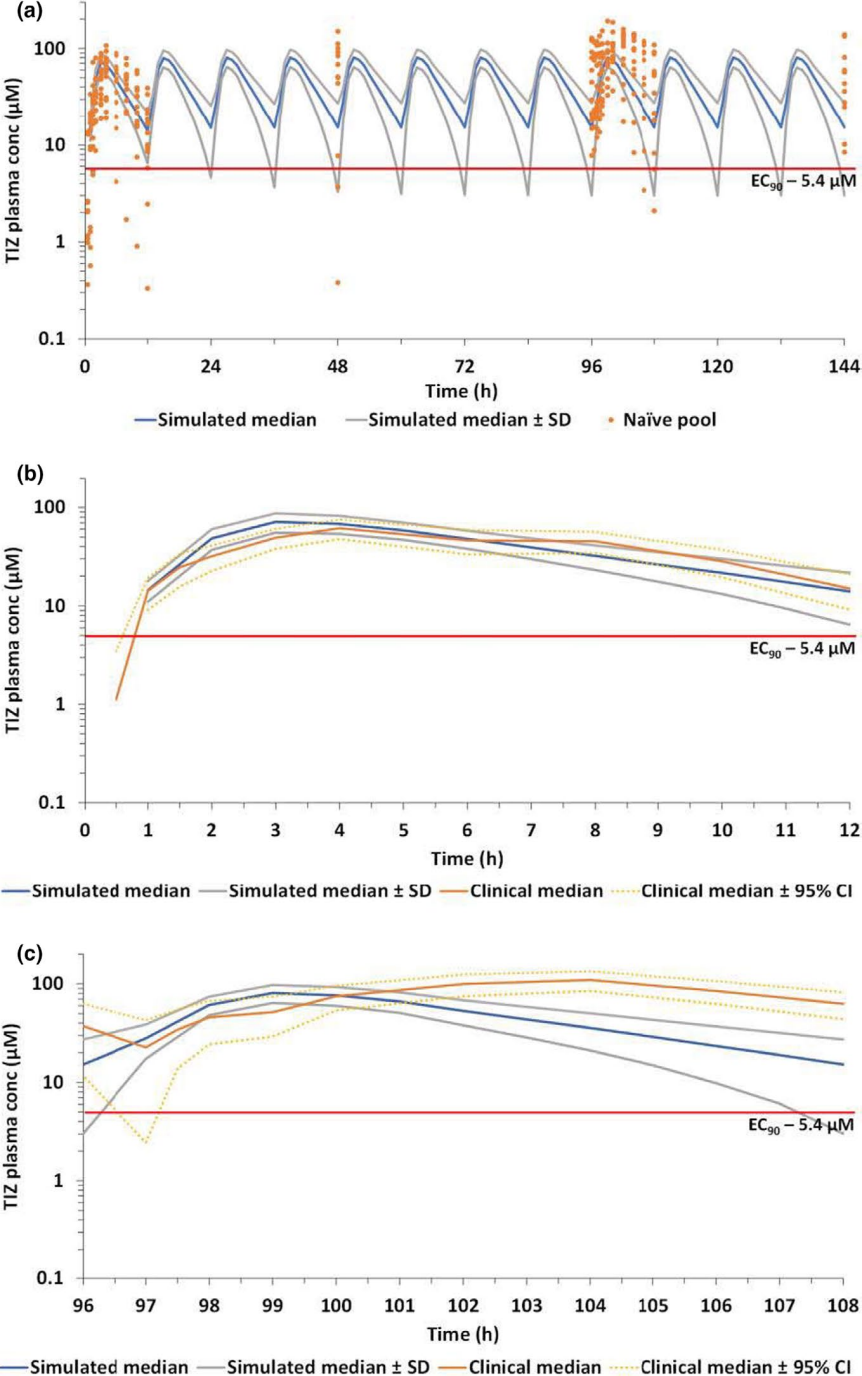
Comparison of PBPK simulated and observed tizoxanide plasma concentrations. (**a**) comparison of median (SD) simulated concentrations (blue) against the naïve pool of plasma concentration in healthy individuals. (**b**) Comparison of observed and simulated median (95% CI) tizoxanide plasma concentrations following the first dose. (**c**) Comparison of observed and simulated median (95% CI) tizoxanide plasma concentrations on day 5. The red line represents the *in vitro* derived EC_90_ against SARS‐CoV‐2 (1.43 mg/L; 5.4 µm). CI, confidence interval; EC_90_, effective concentration 90%; PBPK, physiologically‐based pharmacokinetic; SARS‐CoV‐2, severe acute respiratory syndrome‐coronavirus 2; TIZ, tizoxanide. [Colour figure can be viewed at wileyonlinelibrary.com]

Median C_trough_ at the end of the first dose were above the *in vitro*‐defined target concentration (EC_90_ – 1.43 mg/L) and remained so throughout dosing. The simultaneous fitting of the parent‐metabolite PK model to the tizoxanide and tizoxanide glucuronide datasets is illustrated in **Figure** **2**, with parameter estimates and relative standard errors in **Table** **4**. An acceptable description of the data was obtained with acceptable precision of estimates. Interindividual variability in plasma exposure appears relatively wide but may reflect to some extent variability in administration times for doses taken by patients at home compared with nominal dosing times. The PBPK simulated tizoxanide plasma concentrations relative to the naïve pool of data from healthy individuals in this study is shown over the 7 days of dosing in **Figure** **3a**. **Figure** **3b and c** show the comparison of the observed and simulated median PK profiles on day 1 and day 5, respectively. The corresponding PK data and a numerical comparison between observed and simulated C_max_ and C_trough_ is provided as **Table** **S4**. The trial confirmed prior PBPK predictions for the first dose but with underprediction of exposures at day 5, with higher PK exposures and delayed T_max_ observed clinically than predicted by the PBPK modelling. Because the PBPK model was validated against the clinical data for multiple b.i.d. doses of 500 mg and 1 g nitazoxanide,^26^ where no drug accumulation was observed as the days progressed, the model was not able to capture appropriately the drug accumulation observed with the 1,500 mg b.i.d. regimen with an increasing C_trough_ from day 1 to day 5 and day 7. However, concentrations above the target (i.e., EC_90_ – 1.43 mg/L) were achieved on the first dose and safely maintained throughout the course.

**Table 4:** Parameter estimates for simultaneous parent‐metabolite PK model fitting to tizoxanide and tizoxanide glucuronide plasma concentration data

	CL_TIZ_/*F*_TIZ_, L/h	*V*_TIZ_/*F*_TIZ_, L	*K*_a_, h^−1^	CL_MET_/(*F*_TIZ_*F_MET_), L/h	*V*_MET_/(*F*_TIZ_*F_MET_), L/h
Parameter estimate	3.95	64.99	0.45	5.88	12.71
% RSE*	3.64	8.15	14.20	3.70	12.67

CL_MET_, metabolite clearance; CL_TIZ_, tizoxanide clearance; F_MET_, bioavailability of tizoxanide; F_TIZ_, tizoxanide glucoronide; *K*_a_, absorption rate constant; PK, pharmacokinetic; RSE, relative standard error.

## DISCUSSION

This study establishes the safety and acceptable tolerance of high‐dose nitazoxanide with food in healthy adult participants. A nitazoxanide dose of 1,500 mg twice‐daily for 7 days is safe and the plasma concentrations attained are expected to be sufficient to achieve the *in vitro*‐defined EC_90_ against SARS‐CoV‐2 in over 90% of the population. As expected, GI effects were common (predominantly nausea, abdominal discomfort, bloating, and loose stools) and at most, of moderate intensity (grade 2 discomfort sufficient to cause interference with normal activities). Participants also reported yellow discoloration of sclera and bodily fluids in line with the Summary of Product Characteristics and previous reports, and these fully resolved upon discontinuation of the drug.

Minor elevation in CK, predominantly in the second half of the cohort, was noted in 5 of 14 participants. This was also self‐resolving and not associated with muscle tenderness on physical examination. We believe increased self‐reported physical activity of participants, coinciding with COVID‐19 lockdown restrictions easing, may explain the mild CK elevations observed but ongoing monitoring of CK will be undertaken as part of phase Ib in patients with COVID‐19.

After review, the SRC and the AGILE Independent Data Monitoring and Ethics Committee agreed that these AEs were minor. The POCRM model suggested an escalation to the asymmetric regimen was safe. However, the preferred starting regimen of 1,500 mg b.i.d. was on course to yield optimal exposure and was recommended to progress to evaluation in patients with COVID‐19 and as such the other two potential dosing cohorts did not proceed in this study. An AGILE phase 1b study is now being initiated in South Africa for confirmatory PK analysis and tolerability in patients with COVID‐19, including alternative dosing regimens if required, with seamless transition into phase IIa.

Selection of the target plasma concentration was based upon an *in vitro* derived EC_90_ value generated in Vero cells using nitazoxanide and not tizoxanide.^33^ However, further *in vitro* experimentation was conducted internationally because this original report has demonstrated similar activity in lung epithelial cell models as well as activity of tizoxanide comparable to nitazoxanide itself.^34^, ^35^, ^36^ Furthermore, the antiviral activity has been confirmed to involve inhibition of maturation of the SARS‐CoV‐2 spike protein,^21^ which is similar to the mechanism of antiviral activity for influenza.^37^ More recently, a separate and distinct mechanism involving blocking of spike‐mediated syncytia formation via interactions with TMEM16 has been reported, which may moderate disease severity in parallel to the antiviral effect.^24^ Target concentrations for this secondary mechanism are yet to be clarified but warrant further investigation.

The authors highlight the uncertainty around the therapeutic target concentration for tizoxanide that specifically relates to the impact of plasma protein binding. Consensus on application of protein binding assessments to purported SARS‐CoV‐2 antivirals was published recently in a series of papers in the journal.^38^, ^39^, ^40^ It should be noted that drug binding *in vitro* is almost never zero because drugs bind to culture plastics or serum proteins present in the culture media, even small amounts of culture serum can sometimes bind large amounts of drug, and protein binding may be low affinity/high capacity or vice versa for different drugs. Therefore, empirically determined protein‐adjusted activity values are critical to ascertain the compound‐specific importance of protein binding but are not currently available for nitazoxanide.

Although defining the optimal dose and duration of repurposed therapeutics is fundamental to later trial success, it cannot be assumed that effective doses and exposures directly translate into patients with COVID‐19, particularly those with altered pathophysiology due to severe illness. This is a limitation of all first‐in‐human healthy volunteer studies, within which it is always difficult to generate pharmacodynamic data. The median age and lack of medical comorbidities in this healthy volunteer cohort also differs from the main target population for early use of antivirals, although their use in post‐exposure prophylaxis and to reduce isolation requirements in low‐risk groups is also being considered.

There is an urgent unmet need for safe and effective antiviral therapeutics in early‐stage mild/moderate COVID‐19. These are aimed at preventing progression of disease to hospitalization and death, and possibly also reducing viral transmission in community settings. Repurposing and dose escalation of nitazoxanide for COVID‐19 is supported by *in vitro* data, PBPK modeling and now robust safety and PK data at the 1,500 mg b.i.d. dose. This dose will provide the maximum potential to demonstrate antiviral activity of nitazoxanide in subsequent trials to provide a definitive outcome on the utility of this drug in COVID‐19.

## FUNDING

This trial was funded by Unitaid.

## CONFLICTS OF INTEREST

A.O. is Director of Tandem Nano Ltd. A.O. has received research funding from ViiV, Merck, Janssen and consultancy from Gilead, ViiV, and Merck not related to the current paper. Ridgeback and GlaxoSmithKline have provided funding to the AGILE phase I/II platform to evaluate SARS‐CoV‐2 candidates independently of the current trial. G.G. has received funding from Janssen‐Cilag, Astra Zeneca, Novartis, Astex, Roche, Heartflow, Celldex, BMS, BionTech, Cancer Research UK, NIHR, British Lung Foundation, Unitaid, and GSK for unrelated academic clinical trials and program funding. S.K. has received funding from Merck, ViiV, Janssen, and Gilead for unrelated academic trials. All other authors declared no competing interests for this work.

## AUTHOR CONTRIBUTIONS

S.K., G.G., T.J., P.M., D.L., M.J., R.J.F., G.S., S.E., T.F., A.O., and L.W. designed the research. S.K., G.G., T.J., S.E., G.S., K.T., P.M., H.P., and M.L. analyzed the data. L.W., R.L., R.C., T.R., T.F., R.J.F., M.F., E.O., K.F., R.W., L.J., C.H., S.K., L.E., S.D.P., A.A., H.R., C.W., K.M., I.E., P.M., A.O., R.R., and H.P. performed the research. L.W., T.F., S.K., A.O., and G.S. wrote the manuscript.

## Supporting information

Figure S1

Figure S2

Figure S3

Table S1

Table S2

Table S3

Table S4

Table S5

Supplementary Material
