# Supplementary material for: An Open Label, Adaptive, Phase 1 Trial of High‐Dose Oral Nitazoxanide in Healthy Volunteers: An Antiviral Candidate for SARS‐CoV‐2
Source: Clin Pharmacol Ther. 2021 Nov 13;111(3):585–94. doi: 10.1002/cpt.2463 (PMC8653087; doi:10.1002/cpt.2463)
Supplement: Supplementary file 1 — Figure S1 [file CPT-111-585-s006.pdf]

Figure S1: Schematic and equations of PK model for Tizoxanide and Tixoxanide-Glucuronide plasma concentrations, fitted to observed exposure data.

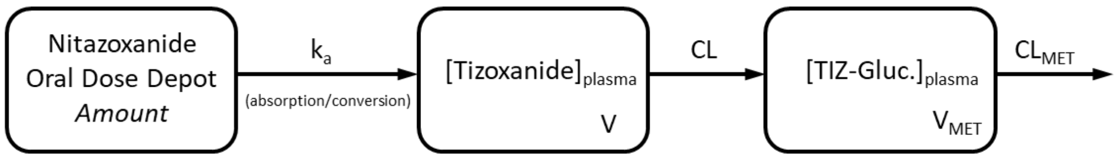

$$d(NTZ_{Depot})/dt = -\ ka * NTZ_{Depot}$$

$$d([Tizoxanide]_{plasma})/dt = +\ ka * NTZ_{Depot}/V \ - CL * [Tizoxanide]_{plasma}/V$$

$$d([TIZ\text{-}Gluc.]_{plasma})/dt = +\ CL * [Tizoxanide]_{plasma}/V_{MET} - CL_{MET} * [TIZ\text{-}Gluc.]_{plasma}/V_{MET}$$

[ ] denotes concentration. ***Apparent*** CL and V parameters estimated as follows, given unknown bioavailability and fraction of total CL accounted for by this metabolic route: $CL = CL/F_{TIZ}$, $V = V/F_{TIZ}$, $CL_{MET} = CL_{MET}/(F_{MET}*F_{TIZ})$, $V_{MET} = V_{MET}/(F_{MET}*F_{TIZ})$
