# Supplementary material for: An Open Label, Adaptive, Phase 1 Trial of High‐Dose Oral Nitazoxanide in Healthy Volunteers: An Antiviral Candidate for SARS‐CoV‐2
Source: Clin Pharmacol Ther. 2021 Nov 13;111(3):585–94. doi: 10.1002/cpt.2463 (PMC8653087; doi:10.1002/cpt.2463)

Figure S2. 12-lead electrocardiogram taken 12-hours following second dose of 1500mg nitzaoxanide in participant 6 displaying artefactual prolonged QTCb with U on T phenomenom.

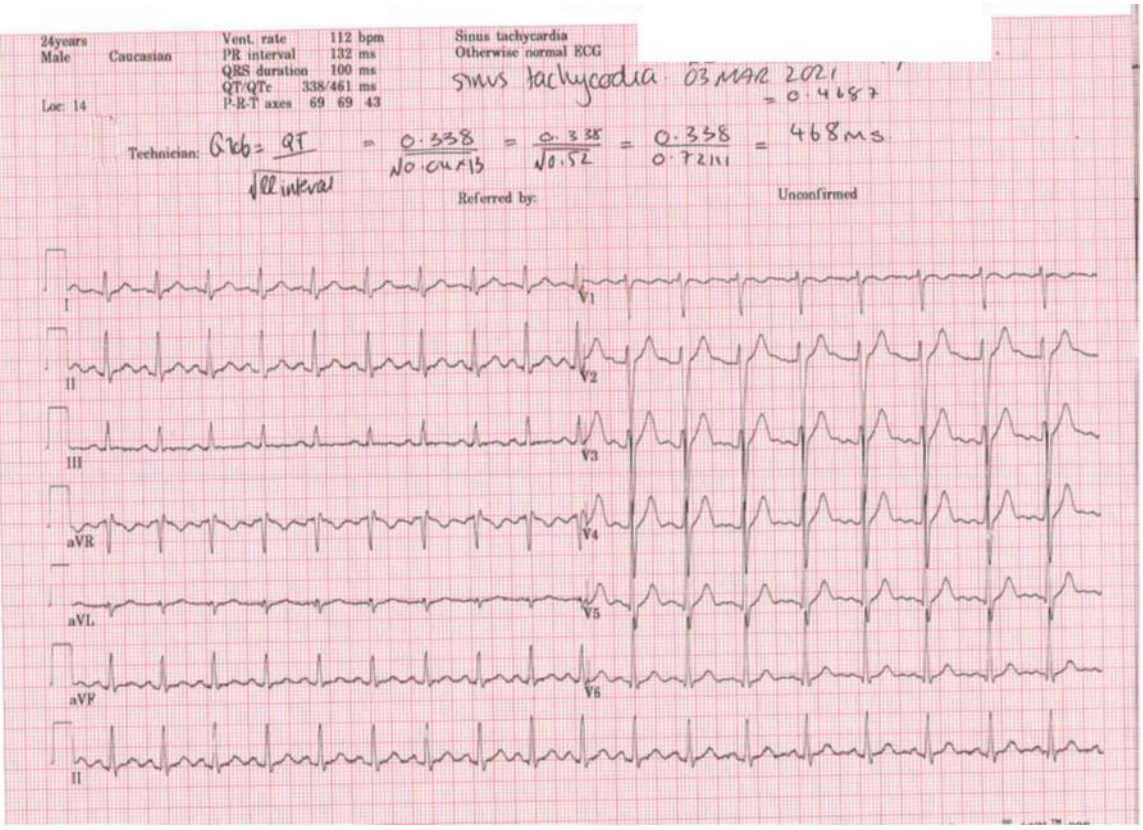

Supplement: Supplementary file 2 — Figure S2 [file CPT-111-585-s005.pdf]
