# Supplementary material for: An Open Label, Adaptive, Phase 1 Trial of High‐Dose Oral Nitazoxanide in Healthy Volunteers: An Antiviral Candidate for SARS‐CoV‐2
Source: Clin Pharmacol Ther. 2021 Nov 13;111(3):585–94. doi: 10.1002/cpt.2463 (PMC8653087; doi:10.1002/cpt.2463)

**Supplementary figure 3**

Comparison of the updated PBPK-simulated and observed TIZ plasma concentrations. A) comparison of median (95% CI) simulated concentrations (blue) against the naïve pool of plasma concentrations in healthy individuals. B) Comparison of observed and simulated median (95% CI) TIZ plasma concentrations following the first dose. C) Comparison of observed and simulated median (95% CI) TIZ plasma concentrations on day 5. The red line represents the in vitro derived EC_90_ against SARS-CoV-2 (1.43 mg/L; 5.4 micromolar).


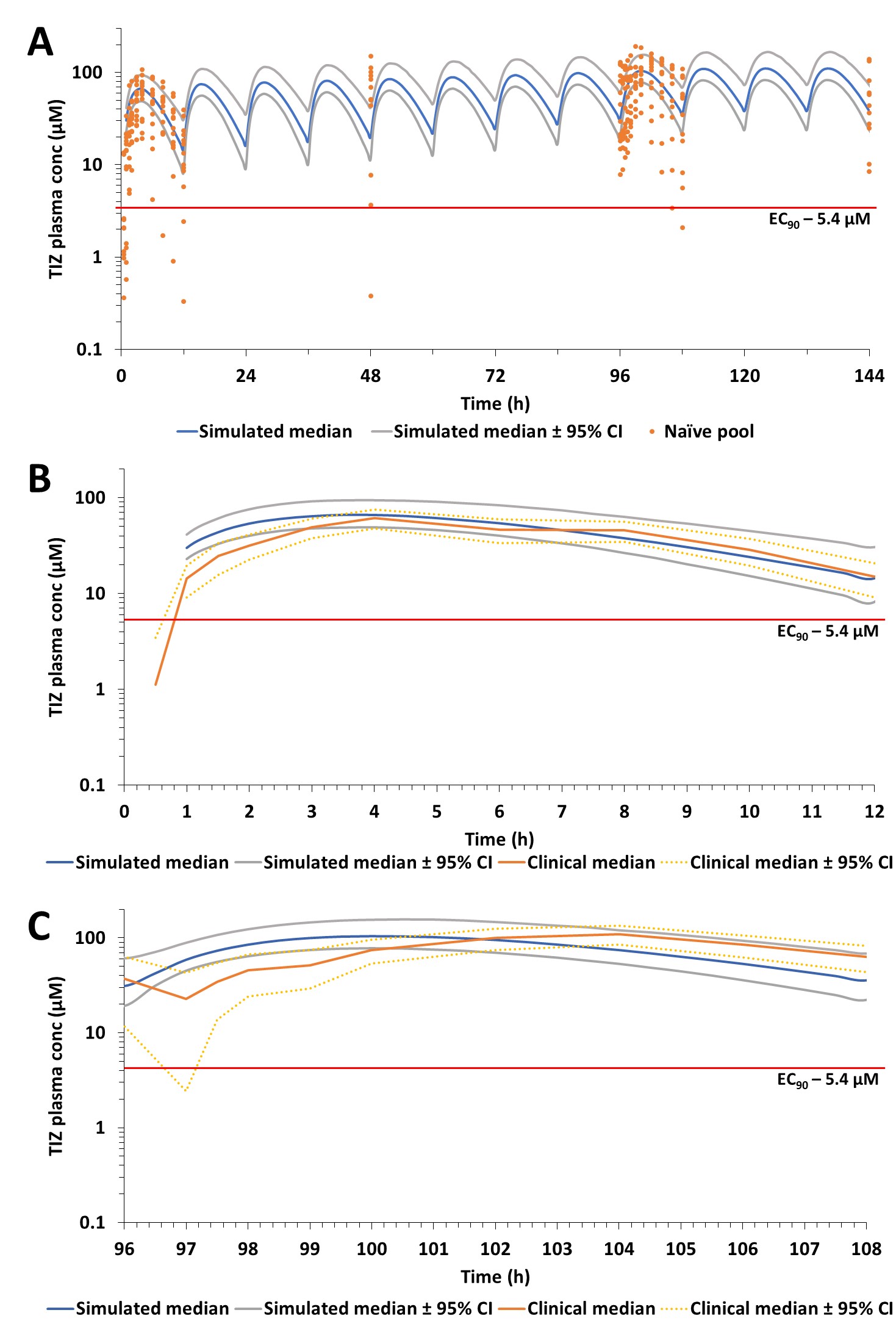

Supplement: Supplementary file 3 — Figure S3 [file CPT-111-585-s004.docx]
