# Supplementary material for: An Open Label, Adaptive, Phase 1 Trial of High‐Dose Oral Nitazoxanide in Healthy Volunteers: An Antiviral Candidate for SARS‐CoV‐2
Source: Clin Pharmacol Ther. 2021 Nov 13;111(3):585–94. doi: 10.1002/cpt.2463 (PMC8653087; doi:10.1002/cpt.2463)
Supplement: Supplementary file 5 — Table S2 [file CPT-111-585-s002.docx]

**Supplemental Table 2** - Nitazoxanide input parameters for the PBPK model

| **Parameter** | **Nitazoxanide** | **Tizoxanide** |
| --- | --- | --- |
| Molecular weight | 307.282 [1] | 265.25 [2] |
| ^*^Protein binding | >99% [1] | >99% [3] |
| Log P | 1.63 [1] | 3.2 [2] |
| pKa (acidic) | 8.3 [1] | 6.7 [4] |
| R | 0.55 | 0.55 |
| Number of hydrogen bond donors | 1 [1] | 2 [2] |
| Polar surface area | 114.11 [1] | 136 [2] |
| Apparent permeability (cm/s) | 1.11e-4 [5] | - |
| Apparent clearance (L/h) | - | 19.34 ± 4.97 [6] |
| Volume of distribution (L) | - | 38.68 ± 14.02 [6] |
| Half-life (h) | - | 1.38 ± 0.29 [6] |

**References**

1. DrugBank. *Nitazoxanide*. 2020 [cited 2020 17/04/2020]; Available from: <https://www.drugbank.ca/drugs/DB00507>.

2. Pubchem. *Tizoxanide*. 2020 [cited 2020 24/04/2020]; Available from: <https://pubchem.ncbi.nlm.nih.gov/compound/Tizoxanide>.

3. Drugs.com. *Nitazoxanide*. 2020 [cited 2020 18/04/2020]; Available from: <https://www.drugs.com/ppa/nitazoxanide.html>.

4. Shalan, S., J.J. Nasr, and F. Belal, *Determination of tizoxanide, the active metabolite of nitazoxanide, by micellar liquid chromatography using a monolithic column. Application to pharmacokinetic studies.* Analytical Methods, 2014. **6**(21): p. 8682-8689.

5. Matysiak-Budnik, T., F. Mégraud, and M. Heyman, *In-vitro transfer of nitazoxanide across the intestinal epithelial barrier.* Journal of Pharmacy and Pharmacology, 2002. **54**(10): p. 1413-1417.

6. Marcelín-Jiménez, G., et al., *Development of a method by UPLC–MS/MS for the quantification of tizoxanide in human plasma and its pharmacokinetic application.* Bioanalysis, 2012. **4**(8): p. 909-917.
