# Supplementary material for: An Open Label, Adaptive, Phase 1 Trial of High‐Dose Oral Nitazoxanide in Healthy Volunteers: An Antiviral Candidate for SARS‐CoV‐2
Source: Clin Pharmacol Ther. 2021 Nov 13;111(3):585–94. doi: 10.1002/cpt.2463 (PMC8653087; doi:10.1002/cpt.2463)
Supplement: Supplementary file 8 — Table S5 [file CPT-111-585-s008.docx]

**Supplemental Table 5** - Tizoxanide pharmacokinetic parameters of observed and predicted data

|  | **Parameter** | **Day 1 (0-12 h)** | **C_48h_** | **Day 5 (96-108 h)** | **C_144h_** |
| --- | --- | --- | --- | --- | --- |
| **Observed PK** | **C_max_ (µM)** | 61.5 (47.6 – 75.3) | - | 109.4 (84.8 – 134) | - |
|  | **C_min_ (µM)** | 14.9 (9.13 – 20.7) | 51.1 (27.7 – 74.5) | 37.3 (11.7 – 62.9) | 43.8 (20.3 – 67.3) |
|  | **AUC (µM.h)** | 509.9 (379.6 – 640.9) | - | 995.5 (718.6 – 1272.4) | - |
|  | **T_1/2_ (h) (apparent, flip flop)** | 5.0 – 6.0 | - | 4.0 – 6.0 | - |
|  | **T_max_ (h)** | 4.0 | - | 6.0 | - |
| **Predicted PK** | **C_max_ (µM)** | 66.4 (48.5 – 93.2) | - | 104.1 (78.3 – 154.8) | - |
|  | **C_min_ (µM)** | 14.4 (8.14 – 30.3) | 19.7 (11.3 – 41.2) | 35.6 (22.2 – 68.2) | 38.2 (23.9 – 73.0) |
|  | **AUC (µM.h)** | 500.4 (358.6 – 768.5) | - | 914.5 (666.9 – 1448) | - |
|  | **T_1/2_ (h) (apparent, flip flop)** | 5.0 – 6.0 | - | 5.0 – 6.0 | - |
|  | **T_max_ (h)** | 3.5 | - | 4.0 | - |
| **Observed vs. Predicted** | **C_max_ (fold change, Obs. vs. PBPK** | 1.03 | - | 1.05 | - |
|  | **C_min_ (fold change, Obs. vs. PBPK)** | 0.93 | 2.59 | 1.04 | 1.15 |
|  | **AUC (fold change, Obs. vs. PBPK)** | 1.02 | - | 1.09 | - |

‘-‘ data not applicable, Cmax, Cmin and AUC are represented as median (95% CI) where available, T_1/2_ is represented as range
